# 3D Printed Wesselsite Nanosheets Functionalized Scaffold Facilitates NIR‐II Photothermal Therapy and Vascularized Bone Regeneration

**DOI:** 10.1002/advs.202100894

**Published:** 2021-08-15

**Authors:** Chen Yang, Hongshi Ma, Zhiyong Wang, Muhammad Rizwan Younis, Chunyang Liu, Chengtie Wu, Yongxiang Luo, Peng Huang

**Affiliations:** ^1^ Marshall Laboratory of Biomedical Engineering International Cancer Center Laboratory of Evolutionary Theranostics (LET) School of Biomedical Engineering Shenzhen University Health Science Center Shenzhen 518060 China; ^2^ Wenzhou Institute University of Chinese Academy of Sciences Wenzhou Zhejiang 325000 China; ^3^ State Key Laboratory of High Performance Ceramics and Superfine Microstructure Shanghai Institute of Ceramics Chinese Academy of Sciences 1295 Dingxi Road Shanghai 200050 China

**Keywords:** angiogenesis, NIR‐II, osteogenesis, photothermal therapy, vascularized bone regeneration

## Abstract

Various bifunctional scaffolds have recently been developed to address the reconstruction of tumor‐initiated bone defects. Such scaffolds are usually composed of a near‐infrared (NIR) photothermal conversion agent and a conventional bone scaffold for photothermal therapy (PTT) and long‐term bone regeneration. However, the reported photothermal conversion agents are mainly restricted to the first biological window (NIR‐I) with intrinsic poor tissue penetration depth. Also, most of these agents are non‐bioactive materials, which induced potential systemic side toxicity after implantation. Herein, a NIR‐II photothermal conversion agent (Wesselsite [SrCuSi_4_O_10_] nanosheets, SC NSs) with tremendous osteogenic and angiogenic bioactivity, is rationally integrated with polycaprolactone (PCL) via 3D printing. The as‐designed 3D composite scaffolds not only trigger osteosarcoma ablation through NIR‐II light generated extensive hyperthermia, but also promote in vitro cellular proliferation and osteogenic differentiation of rat bone marrow mesenchymal stem cells (rBMSCs) and human umbilical vein endothelial cells (HUVECs), respectively, and the ultimate enhancement of vascularized bone regeneration in vivo owing to the controlled and sustained release of bioactive ions (Sr, Cu, and Si). The authors' study provides a new avenue to prepare multifunctional bone scaffolds based on therapeutic bioceramics for repairing tumor‐induced bone defects.

## Graphical abstract

An efficient multifunctional bone scaffold based on 3D printed SrCuSi_4_O_10_ nanosheets/polycaprolactone composite is developed as SC/PCL, which possesses excellent therapeutic effects for deep‐seated osteosarcoma due to its high photothermal‐conversion efficiency in the second biological window. Moreover, the as‐designed 3D composite scaffold could facilitate the vascularized bone regeneration via the sustained release of bioactive ions (Sr, Si, and Cu).

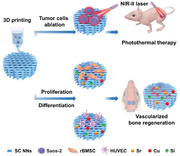

## 1 Introduction

Bone related malignancies, including primary bone cancer (osteosarcoma, chondrosarcoma, fibrosarcoma, etc.) and bone metastatic cancer (primary tumors in breast, lung, kidney, etc.) seriously threaten the patient's survival.^[^ ^1^ ^]^ Although surgical treatment combined with adjuvant chemotherapy in the clinic has substantially promoted the survival outcomes, an incomplete surgical tumor resection, aberrant side effects of chemotherapy, and unfortunate cancer invasiveness, hold a great possibility to evoke cancer recurrence or metastasis. Besides, bone defects associated with the surgical interventions are almost inevitable, which usually require bone grafts to guide new bony ingrowth.^[^ ^2^ ^]^ Thus, it is highly crucial to develop multifunctional implants, which not only possess remarkable bone remodeling capacity, but also enable the eradication of residual tumor cells.

Recently, tremendous efforts have been made to exploit bifunctional bone grafts to address the challenging tumor‐induced bone defects.^[^ ^1^, ^3^ ^]^ The design of a conventional bone graft is usually based on the integration of a typical bone scaffold with a photothermal agent via surface coating. Briefly, under NIR light excitation, the surface‐coated photothermal agent induces PTT because of the generation of localized hyperthermia, while the bone scaffold offers the long‐term support for bone repairing. For example, Pan et al. demonstrated the fabrication of a composite scaffold by integrating 3D printed bioactive glass (BG) scaffold with 2D Ti_3_C_2_ nanosheets,^[^ ^1c^ ^]^ facilitating simultaneous NIR‐I activated PTT of osteosarcoma and the stimulation of new bone formation in vivo. Similar composite scaffolds such as graphene oxide coated *β*‐tricalcium phosphate scaffolds and polydopamine coated Nagel (Ca_7_Si_2_P_2_O_16_) scaffolds were also developed by our group, previously.^[^ ^1^, ^2^ ^]^ Though these composite scaffolds exhibited positive bone repairing and anti‐tumor capacity, the ultimate clinical applications remain a formidable challenge because of their absorption in the NIR‐I region.^[^ ^4^ ^]^ Compared to the intrinsic limitations like poor laser light penetration (≈1–2 cm) and maximum permissible exposure (0.33 W cm^−2^) of NIR‐I biowindow (NIR‐I, 650–1000 nm), NIR‐II biowindow (1000‐1350 nm) with much deeper light penetration (>2 cm) and remarkably higher MPE (1 W cm^−2^) is more attractive for biomedical applications.^[^ ^5^ ^]^ Thus, it is imperative to develop NIR‐II photothermal agent functionalized bone scaffolds to treat tumor‐induced bone defects.

Strontium copper tetrasilicate (SrCuSi_4_O_10_, denoted as SC), which belongs to the “Egyptian Blue Family” (*X*CuSi_4_O_10_, *X* represents Ca, Sr, or Ba), has been recently exfoliated into nanosheets (NSs) due to its inherent layered structure similar to other 2D materials.^[^ ^6^ ^]^ The exfoliated SC NSs possessed excellent photothermal conversion efficiency (≈46.3%) in the NIR‐II region and high biocompatibility both in vitro and in vivo, indicating the possibility of SC NSs as NIR‐II photothermal agent for bone tumor ablation.^[^ ^6b^ ^]^ It is notable to mention that Si or Sr‐containing biomaterials have already been well‐studied as bone scaffolds due to the sustained release of Si and Sr ions, which have been proved as the two main powerful elements for osteogenesis.^[^ ^7^ ^]^ Moreover, recent studies demonstrated that CaCuSi_4_O_10_ could be used for tissue reconstruction as the released Cu, Sr or Si ions stimulate new blood vessel formation by expediting the expression of hypoxia‐inducible factor‐1 (HIF‐1*α*) and vascular endothelial growth factor (VEGF).^[^ ^8^ ^]^ It is reasonable to conceive that SC NSs might hold great prospects as NIR‐II photothermal agents with enhanced vascularized bone regeneration property.

**Figure 1:**
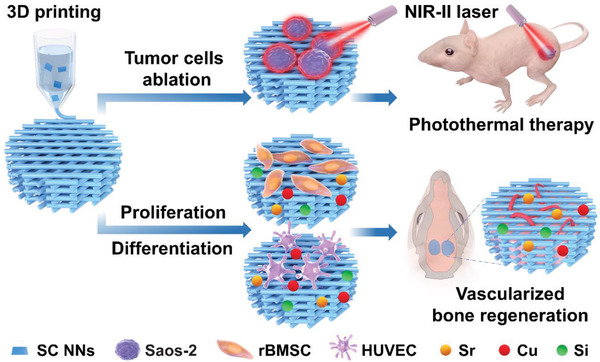
Schematic illustration of 3D printed SC/PCL composite scaffolds for bone tumor photothermal eradication and vascularized bone regeneration, respectively.

To ensure the possible clinical use of SC NSs, an FDA (US Food and Drug Administration) approved polymer, polycaprolactone (PCL) is utilized to incorporate with SC NSs. PCL has been widely developed as 3D printed scaffolds for biomedical applications such as bone tissue engineering due to its outstanding biocompatibility and superior rheological/viscoelastic properties, however, an inherent bioinert character hinders its potential applications as a bone scaffold. Combining PCL with bioactive ceramics has been a common strategy for preparing bone scaffolds. Herein, 3D printed SC NSs/polycaprolactone (SC/PCL) composite scaffolds were prepared as a bifunctional therapeutic implant for bone tumor photothermal eradication and vascularized bone regeneration (**Figure** **1**). On one hand, osteosarcoma could be ablated by the hyperthermia generated from the photothermal conversion ability of SC NSs under NIR‐II light. On the other hand, SC/PCL composite scaffolds could promote the osteogenic differentiation of rat bone marrow mesenchymal stem cells (rBMSCs) and angiogenic differentiation of human umbilical vein endothelial cells (HUVECs) in vitro, following the stimulation of vascularized bone reconstruction in vivo due to the sustained release of bioactive ions (Sr, Cu, and Si). Such a proof‐of‐concept study provides promising new avenues to develop multifunctional bone scaffolds for repairing tumor‐induced bone defects.

## 2 Results and Discussion

### 2.1 Fabrication and Characterization of 3D Printed SC/PCL Composite Scaffolds

**Figure 2:**
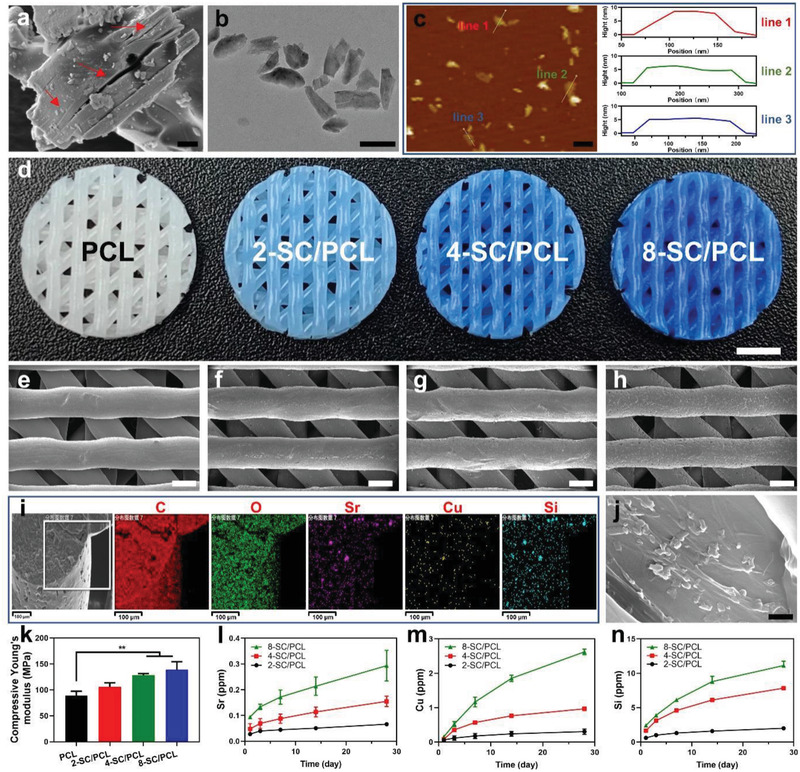
Characterization of SC NSs and 3D printed SC/PCL composite scaffolds. a,b) SEM images of SC bulk (a) and exfoliated SC NSs (b). c) AFM image and height profile of SC NSs. d) Optical images of 3D printed PCL and SC/PCL composite scaffolds. e–h) SEM characterization of macroporous structures of PCL (e), 2‐SC/PCL (f), 4‐SC/PCL (g), and 8‐SC/PCL (h) scaffolds. i) EDS elemental mapping analysis of SC NSs in 3D printed 4‐SC/PCL composite scaffold. j) SEM images of the cross‐section view of 3D printed 4‐SC/PCL composite scaffold. k) Compressive Young's moduli of different scaffolds. l–n) Ion release curves of Sr (l), Cu (m), and Si (n) ions from various composite scaffolds. Scale bar: 1µm (a), 200 nm (b,c), 2 mm (d), 500 µm (e–h), and 500 nm (j). Data are presented as mean ± s.d. (k) (*n* = 3), (l–n) (*n* = 4). ** For 0.001 < *p* < 0.01. One‐way ANOVA analysis.

SC bulk powders were successfully synthesized following a conventional solid‐state reaction method as the crystal structure was identified by X‐ray diffraction (XRD) patterns (Figure S1, JCPDS Card No. 49–1813, Supporting Information). The as‐developed SC bulk powders exhibited multilayer microstructures (**Figure** **2**a, red arrow), which were ultrasonically exfoliated into SC NSs in deionized water (Figure 2b). The delaminated SC NSs were about 200 nm in size with a thickness of ≈5–10 nm (Figure 2c), as determined by the atomic force microscope (AFM), demonstrating the successful fabrication of ultrathin 2D SC NSs.

Bone scaffolds are usually prepared with interconnected pores, which are essential for cells ingrowth and the transport of nutrition.^[^ ^9^ ^]^ Despite several conventional methods have been developed to prepare porous scaffolds such as gas foaming technique and polyurethane foam templating method, it is quite hard to accurately control the pore structures using these methods.^[^ ^10^ ^]^ The recently developed 3D printing technique has shown distinct advantages in preparing porous bone scaffolds as all the pore parameters could be precisely designed by computer‐assisted design (CAD).^[^ ^7^, ^11^ ^]^ More importantly, by the combination of scanning software with 3D printing techniques, manufacturing of customized bone scaffolds are highly expected for irregular bone defects. Here, SC/PCL composite scaffolds with various concentrations of SC NSs (2, 4, and 8 wt%, denoted as 2‐SC/PCL, 4‐SC/PCL, and 8‐SC/PCL) were further fabricated through an extrusion‐based 3D printing technique. The color of the resultant scaffolds turned dark with the increase of the incorporated SC NSs as shown in the optical images (Figure 2d). Scanning electron microscope (SEM) revealed that all the scaffolds possessed smooth surface (Figure S2, Supporting Information) with uniform 45° interlaced architectures (Figure 2e–h), indicating the encapsulation of SC NSs into the PCL matrix. Energy‐dispersive X‐ray spectroscopy (EDS) elemental mapping revealed the presence of Sr, Cu, Si, and O element (Figure 2i), confirming the successful encapsulation of SC NSs. For the direct observation of encapsulated SC NSs, the composite scaffold was physically cut in liquid nitrogen. As shown in Figure 2j, the encapsulated SC NSs could be easily recognized (red arrow). Despite the controllable porous structures, 3D printed scaffolds usually show a better mechanical property than the scaffolds fabricated via conventional methods.^[^ ^7a^ ^]^ In this study, although there was no significant difference of compressive strength between different scaffolds (Figure S3, Supporting Information), the compressive Young's moduli were obviously higher in composite scaffolds. Compared to pure PCL scaffolds, the incorporation of the SC NSs significantly improved the compressive Young's moduli of 3D printed SC/PCL composite scaffolds (Figure 2k), while the 8‐SC/PCL group exhibited the highest Young's modulus of 138.89 ± 15.25 Mpa, suggesting that inorganic nanosheets could remarkably enhance the Young's modulus of the scaffolds. However, the high content of SC NSs (8 wt%) negatively affected the tensile mechanical property as the tensile strength and toughness of 8‐SC/PCL composite scaffolds were significantly lower than pure PCL scaffolds (Figures S4 and S5, Supporting Information), which may be ascribed to the interruption of nanosheets on PCL network. Furthermore, the accelerated degradation of the scaffolds was evaluated by soaking scaffolds in 5 m NaOH solution. As shown in Figure S6, Supporting Information, composite scaffolds exhibited much faster degradation than pure PCL scaffolds, and 8‐SC/PCL composite scaffold had the fastest degradation rate as it was almost dissolved after 4 days. When using PBS as a treating buffer, the sustained release of bioactive ions (Sr, Cu, Si) was noticed from 3D printed SC/PCL composite scaffold in vitro till 4 weeks (Figure 2l–n), and SC/PCL scaffolds with a high concentration of SC NSs exhibited much higher ionic release. Since the release of ions was accompanied by the degradation of scaffolds, a significant decrease in the compressive mechanical properties of SC/PCL composite scaffold was recorded after degradation for 4 weeks (Table S1, Supporting Information). According to the previous studies, Sr and Si ions play a vital role in osteogenesis, while suitable concentrations of Cu and Si ions can stimulate angiogenesis.^[^ ^7^, ^8^, ^12^ ^]^ Thus, 3D printed SC/PCL composite scaffolds are successfully fabricated and expected to possess enhanced vascularized bone regeneration property.

### 2.2 In Vitro Osteogenic and Angiogenic Performance of 3D Printed SC/PCL Composite Scaffolds

**Figure 3:**
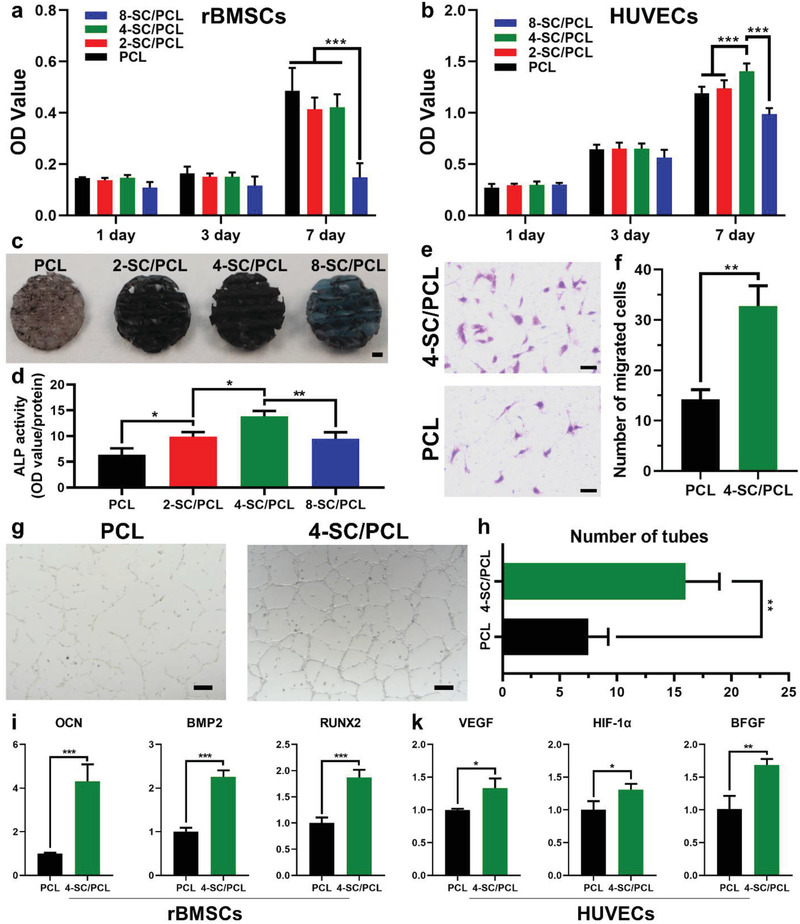
In vitro osteogenesis and angiogenesis assay. a) rBMSCs proliferation on different scaffolds. b) HUVECs proliferation on different scaffolds. c,d) ALP staining (c) and the quantitative analysis (d) of rBMSCs on various scaffolds after cultivation for 7 days. e,f) HUVECs migration analysis using transwell method after co‐cultured with 3D printed PCL and 4‐SC/PCL composite scaffolds. g,h) In vitro tube formation assay of HUVECs after being co‐cultured with 3D printed PCL and 4‐SC/PCL composite scaffolds. i) Osteogenic gene (OCN, BMP2, RUN2) expression of rBMSCs cultured on 3D printed PCL and 4‐SC/PCL composite scaffolds for 7 days. k) Angiogenic gene (VEGF, HIF‐1*α*, BFGF) expression of HUVECs cultured on 3D printed PCL and 4‐SC/PCL composite scaffolds for 7 days. Scale bar: 1 mm (c), 100 µm (e), and 200 µm (g). Data are presented as mean ± s.d. (a,b,f,h,i) (*n* = 4), (d,k) (*n* = 3). * For 0.01 < *p* < 0.05, ** for 0.001 < *p* < 0.01, and *** for *p* < 0.001. One‐way ANOVA analysis.

In vitro osteogenic and angiogenic performance of 3D printed SC/PCL composite scaffolds were evaluated by using rBMSCs and HUVECs, respectively. As shown in **Figure** **3**a,b, both rBMSCs and HUVECs are effectively proliferated on different 3D printed scaffolds, while 4‐SC/PCL scaffolds displayed the best proliferation compared to other groups. It is worth mentioning that 8‐SC/PCL scaffolds exhibited obvious cytotoxicity, which might be ascribed to the extensive‐release of Cu ions (4.04 ppm, Table S2, Supporting Information).^[^ ^13^ ^]^ During the bone mineralization process, alkaline phosphatase (ALP) plays a pivotal role through binding to bone matrix proteins and stimulating pyrophosphate hydrolysis, making it a renowned biomarker of osteogenesis.^[^ ^14^ ^]^ Both the qualitative and quantitative analysis of ALP expression in rBMSCs on different scaffolds were conducted by ALP staining kit and assay kit. As shown in Figure 3c,d, 3D printed composite scaffolds significantly enhanced the ALP expression than 3D printed PCL scaffolds alone, indicating the superior osteogenic bioactivity of composite scaffolds, while 4‐SC/PCL scaffolds hold the highest osteogenic performance. Therefore, we chose 4‐SC/PCL scaffolds as representative composite scaffolds for further cellular studies.

The effect of 4‐SC/PCL scaffolds on HUVEC migration was estimated through the transwell assay (Figure 3e). More HUVECs migrated in the group of 4‐SC/PCL than PCL alone, after cultured for 8 h (Figure 3f). Similarly, the in vitro tube formation assay revealed that more tubes formed in Matrigel co‐cultured with 4‐SC/PCL scaffolds as compared to PCL group (Figure 3g). The corresponding quantitative analysis showed that the number of tubes formed in 4‐SC/PCL was significantly higher (≈2.1 fold) than that in PCL group (Figure 3h), indicating that 4‐SC/PCL scaffolds had a better effect on the pro‐angiogenesis of HUVECs. The quantitative real‐time PCR (qRT‐PCR) assay further revealed the dual osteogenesis (Figure 3i) and angiogenesis (Figure 3k) bioactivity of 4‐SC/PCL scaffolds, because in comparison to PCL scaffolds alone, osteogenic genes expression (osteocalcin [OCN], bone morphogenetic protein‐2 [BMP2], and runt‐related transcription factor 2 [RUNX2]) of rBMSCs and angiogenic genes expression (VEGF, hypoxia‐inducible factor‐1 alpha [HIF‐1*α*], and basic fibroblast growth factor [BFGF]) of HUVECs were significantly up‐regulated on 4‐SC/PCL scaffolds. This might be ascribed to the sustained release of bioactive ions (Sr, Cu, and Si) as a large number of previous studies have proved the osteogenic bioactivity of Sr and Si ions, and the angiogenic bioactivity of Cu and Si ions, respectively.^[^ ^7^, ^8^ ^]^

### 2.3 3D Printed SC/PCL Composite Scaffolds Promote Vascularized Bone Regeneration In Vivo

**Figure 4:**
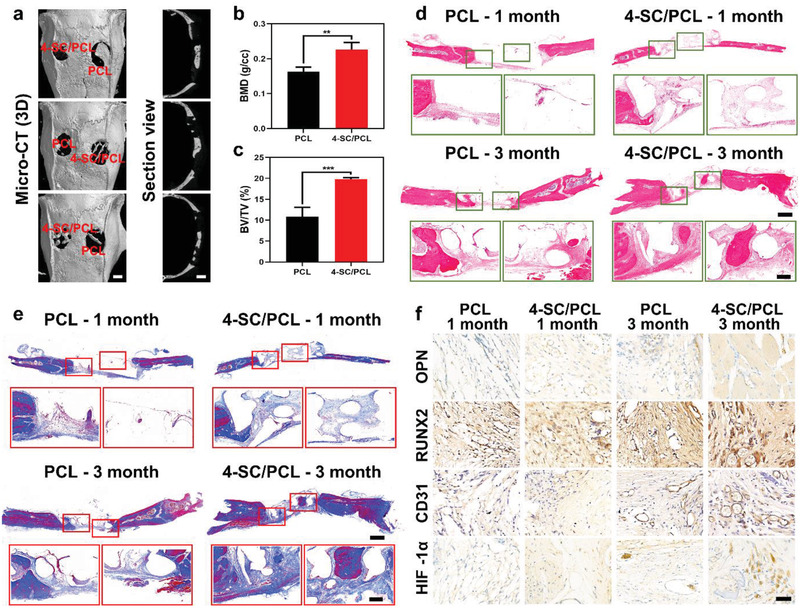
In vivo vascularized bone regeneration. a–c) Typical 3D reconstruction of micro‐CT images (a) and analysis of new bone formation in defect areas after implantation of 3D printed PCL and 4‐SC/PCL composite scaffolds for 3 months: b) bone mineral density (BMD) and c) bone volume/total volume (BV/TV). d,e) Representative H&E staining (d) and Masson's trichrome staining (e) of the craniums with cranial defects after implantation of 3D printed PCL and 4‐SC/PCL composite scaffolds for 1 and 3 months. f) The immunohistochemistry staining targeting OPN, RUNX2, CD31, and HIF‐1*α* in new‐formed tissues after treating with 3D printed PCL and 4‐SC/PCL composite scaffolds for 1 and 3 months. Scale bar: 2 mm (a), 1 mm (low‐magnification images in d and e), 200 µm (high‐magnification images in d and e), 50 µm (f). Data are presented as mean ± s.d. (b,c) (*n* = 4). ** For 0.001 < *p* < 0.01 and *** for *p* < 0.001. One‐way ANOVA analysis.

To further investigate the in vivo osteogenic performance of 3D printed SC/PCL composite scaffolds, a typical rat calvarial defect model was established and implanted with both 3D printed PCL and 4‐SC/PCL scaffolds for 1 and 3 months, respectively. Micro‐CT 3D reconstruction images revealed the deposition of new bone tissues in both PCL and 4‐SC/PCL scaffolds over 3 months as shown in **Figure** **4**a. Whereas, compared to PCL scaffolds alone, quantitative analysis showed 1.39 and 1.82‐fold higher bone mineral density (BMD, Figure 4b) and bone volume/total volume (BV/TV, Figure 4c) in 4‐SC/PCL scaffolds, suggesting that 4‐SC/PCL scaffolds significantly promoted more bone mineralization than PCL scaffolds. The histopathological analysis by hematoxylin and eosin (H&E, Figure 4d) and Masson's trichrome staining (Figure 4e) further demonstrated the time‐dependent growth of more newborn osseous tissues into both PCL and 4‐SC/PCL scaffolds. Meanwhile, no obvious inflammatory cells were observed in either PCL or 4‐SC/PCL group, indicating their good biocompatibility in vivo. It is worthwhile to mention that the better osteogenic activity of 4‐SC/PCL scaffolds could be easily observed as more newly formed bone tissues were grown into the region adjacent to the original bone (left rectangular box) and in the middle of the defect (right rectangular box), which is consistent with the results of the micro‐CT analysis.

The possible reason for the excellent bone regeneration performance of composite scaffolds mainly accounts for the sustained release of bioactive ions (Sr, Cu, and Si) from 4‐SC/PCL scaffold as Sr or Si‐containing biomaterials have been widely reported to facilitate bone healing.^[^ ^7a,b^ ^]^ It has been suggested that Sr could activate the ERK‐MAPK and Wnt signaling to promote osteogenic differentiation, while Si could enhance bone formation by the AMPK/ERK1/2 signaling pathway.^[^ ^15^ ^]^ Interestingly, the combination of the Sr and Si could activate MAPK signaling pathways by the up‐regulation of the ERK and p38 AMPK phosphorylation, resulting in enhanced bone regeneration.^[^ ^16^ ^]^ Moreover, apart from osteogenesis, vascularization also plays a key role in bone reconstruction process via nutrients supply and renewable autologous cells.^[^ ^9^, ^16^ ^]^ As reported, Cu could stabilize HIF‐1*α* and enhance the expression of VEGF, while the combination of Cu and Si ions or the combination of Sr and Si ions could achieve synergistic stimulatory effects on vascularization.^[^ ^8^, ^16^, ^17^ ^]^ Considering the presence of both Cu and Si ions in 4‐SC/PCL scaffold, an accelerated vascularized bone regeneration was highly anticipated. As a proof of concept, immunohistochemical staining against osteogenic biomarkers, including osteopontin (OPN) and RUNX2 and angiogenic biomarkers (platelet endothelial cell adhesion molecule‐1 PECAM‐1, CD31, and HIF‐1*α*) was conducted (Figure 4f). The osteogenic and angiogenic performance of both PCL and 4‐SC/PCL scaffolds were reconfirmed as more OPN, RUNX2, CD31, and HIF‐1*α* expression was recorded at longer time points, whereas 4‐SC/PCL group showed higher OPN expression at each time point as compared to the PCL group. All these results pinned the conclusion that 3D printed 4‐SC/PCL composite scaffolds hold great potential as a bone graft to enhance vascularized bone regeneration.

### 2.4 In Vitro Anticancer Effects of 3D Printed SC/PCL Composite Scaffolds

**Figure 5:**
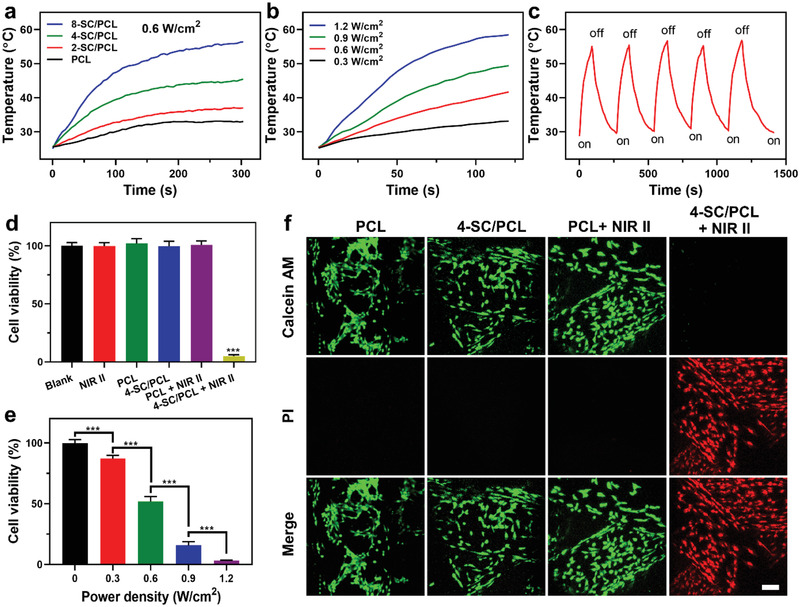
In vitro photothermal antitumor killing activity. a) Photothermal heating (1064 nm, 0.6 W cm^−2^) curves of different 3D printed scaffolds. b) Photothermal heating curves of 3D printed 4‐SC/PCL scaffolds under 1064 nm laser irradiation at varying power densities (0.3, 0.6, 0.9, and 1.2 W cm^−2^). c) Photothermal stability of 3D printed 4‐SC/PCL scaffolds under sequential laser on/off cycles. d) Relative cell viability of Saos‐2 cells after different treatments as described. e) Laser power dependent relative cell viability of Saos‐2 cells treated with 3D printed 4‐SC/PCL scaffolds. f) The calcein AM/PI‐stained images of Saos‐2 cells on 3D printed PCL and 4‐SC/PCL composite scaffolds with/without NIR‐II laser irradiation. Scale bar: 100 µm. Data are presented as mean ± s.d. (d,e) (*n* = 5). *** For *p* < 0.001. One‐way ANOVA analysis.

SC nanosheets with high photothermal conversion efficiency (46.3%) in NIR‐II biological window have been carefully studied in our previous study.^[^ ^6b^ ^]^ Such efficiency was obviously comparative or higher than other reported NIR‐II photothermal agents such as V_2_C quantum dots (45.05%),^[^ ^18^ ^]^ PEGylated Cu_3_BiS_3_ nanorods (40.7%),^[^ ^19^ ^]^ and Cu_3_P Nanocrystals (27%),^[^ ^20^ ^]^ showing great potential as photothermal agents for NIR‐II PTT. The in vitro photothermal performance of 3D printed SC/PCL composite scaffolds was evaluated. A considerable temperature elevation (Δ31.5 °C for 8‐SC/PCL) with a strong dependence on SC concentration was observed in SC/PCL scaffolds under 1064 nm laser irradiation (0.6 W cm^−2^) for 5 min (**Figure** **5**a). As a control, only 7.4 °C temperature enhancement was recorded in PCL scaffold under the same irradiation conditions. Despite the ratio of SC/PCL, the photothermal performance of the composite scaffolds was also dependent on the laser power density as the significant temperature elevation was noticed under higher laser power irradiations (Figure 5b). Moreover, no apparent temperature decrease was found even under 6 repetitive NIR‐II lasers on/off cycles (Figure 5d), while a slight temperature decrease was noticed after in vitro degradation (in PBS) for even 4 weeks (Table S1, Supporting Information), implying the excellent photothermal stability of 4‐SC/PCL scaffolds, which is beneficial for sustained photothermal ablation of bone tumors.

To further validate the anticancer efficacy of 3D printed SC/PCL composite scaffolds, Saos‐2 cells were incubated on PCL and 4‐SC/PCL scaffolds for 24 h and subsequently irradiated by NIR‐II laser irradiation (1 W cm^−2^) for 5 min. As expected, both PCL and 4‐SC/PCL scaffolds did not show any cytotoxicity in dark (Figure 5d). However, under NIR‐II laser excitation, 4‐SC/PCL scaffolds triggered >90% ablation of Saos‐2 cells, which is far superior to the negligible cell death caused by PCL scaffolds alone. On the other hand, under high laser power excitation, 4‐SC/PCL scaffolds exhibited a significant reduction in cellular viability in vitro as shown in Figure 5e. Moreover, calcein‐AM/propidium iodide (PI) staining further endorsed the MTT results as PCL scaffolds showed negligible Saos‐2 cell death under NIR‐II laser irradiation, while 4‐SC/PCL scaffolds triggered complete tumor cells ablation as confirmed by PI staining (Figure 5f). All these results demonstrated the prominent in vitro therapeutic effects of 3D printed SC/PCL composite scaffolds.

### 2.5 In Vivo Antitumor Effects of 3D Printed SC/PCL Composite Scaffolds

**Figure 6:**
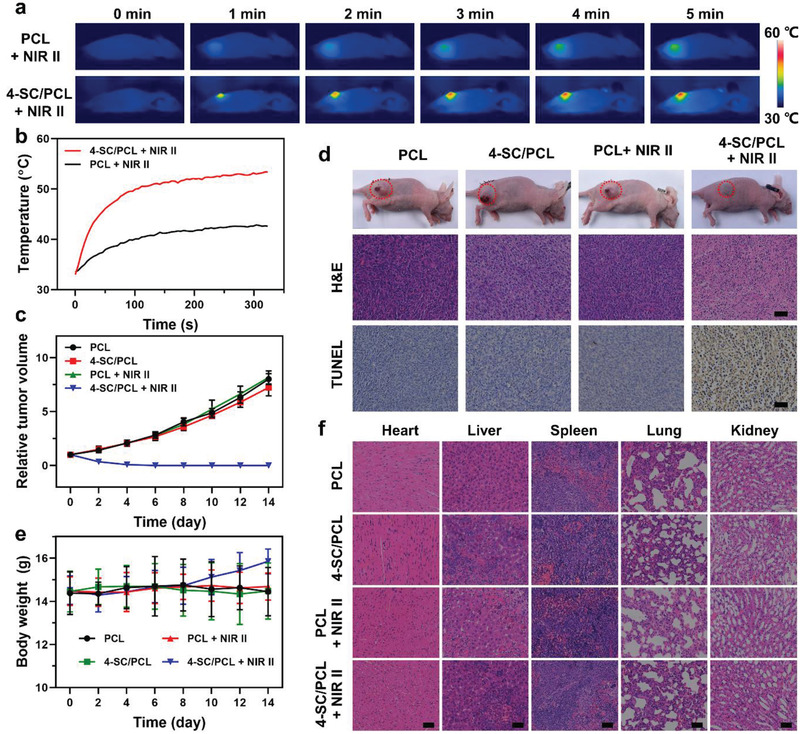
In vivo antitumor efficacy. a,b) Thermal images (a) and the corresponding photothermal heating curves (b) of 3D printed PCL and 4‐SC/PCL composite scaffolds under 1064 nm laser irradiation (1 W cm^−2^, 5 min). c) Relative tumor volume of the indicated different groups after treatments for 14 days. d) Histological analysis (H&E and TUNEL staining) of tumor sections collected from different groups. e) Body weight of the indicated different groups after treatments for 14 days. f) H&E staining of the major organs harvested from different groups. Scale bar: 50 µm. Data are presented as mean ± s.d. (c,e) (*n* = 5).

Encouraged by the excellent in vitro photothermal performance of 3D printed SC/PCL composite scaffolds, the in vivo photothermal antitumor effects were then studied. The Saos‐2 tumor‐bearing mice were prepared and randomly divided into four groups: 1) PCL scaffold; 2) 4‐SC/PCL scaffold; 3) PCL scaffold + 1064 nm laser; and 4) 4‐SC/PCL scaffold + 1064 nm laser. The scaffolds were implanted into the bottom of tumors and the in situ thermal images were recorded by a thermal imaging camera (**Figure** **6**a). The temperature of tumor sites treated with 4‐SC/PCL scaffolds raised rapidly in the first 2 min and then increased slowly for the other 3 min with the maximum temperature of 53.4 °C, enabling high‐temperature PTT in vivo (Figure 6b). In contrast, the maximum temperature of tumor sites treated with PCL scaffold was 42.6 °C under the same laser dose. The relative tumor volumes were calculated every 2 days for 14 days. In contrast to the progressive tumor growth in the control and PCL scaffolds groups, complete tumor eradication was seen in 4‐SC/PCL scaffolds even after 4 days (Figure 6c,d). Hematoxylin and eosin (H&E) and the terminal deoxynucleotidyl transferase uridine triphosphate nick end labeling (TUNEL) staining assays were further performed to determine the antitumor efficacy of the 4‐SC/PCL scaffolds. As shown in Figure 6d, much severe cellular apoptosis was observed in group 4 as compared to other groups, confirming excellent photothermal treatment efficacy of 4‐SC/PCL scaffolds under NIR‐II laser irradiation. Importantly, no particular change was recorded in the mouse body weight during the course of treatment (Figure 6e), while the H&E staining of major organs (Figure 6f), indicated the good biocompatibility of scaffolds. Thus, 3D printed SC/PCL composite scaffolds hold great potential as a safe and efficient antitumor platform due to the outstanding biocompatibility and photothermal performance.

## 3 Conclusions

In summary, a bifunctional platform‐based on 3D printed SC/PCL composite scaffolds for simultaneous NIR‐II photothermal therapy of osteosarcoma and enhanced vascularized bone regeneration was successfully fabricated. The high photothermal‐conversion efficiency of SC NSs in the NIR‐II region endows scaffolds with excellent photothermal therapeutic effects for deep‐seated osteosarcoma without any significant adverse effects. Moreover, the released bioactive ions (Sr, Si, and Cu) could not only facilitate the osteogenic differentiation of rBMSCs and the angiogenic differentiation of HUVECs in vitro, but also enhance the new bone formation with more vessels in vivo. This as‐designed stepwise therapeutics takes full advantage of the multifunctional properties of bioceramics and broadens the avenue to engineer high‐performance therapeutic platform for versatile biomedical applications.

## 4 Experiment Section

### Materials

All the chemicals were purchased from Aladdin Reagent Co., Ltd. (Shanghai, China) except otherwise mentioned. PCL was obtained from J&K Scientific Ltd. (Shanghai, China) and Matrigel was bought from BioSciences (CA, USA).

### Fabrication of SC NSs

SC powders were prepared according to a previous study.^[^ ^21^ ^]^ Briefly, silicon dioxide (SiO_2_), strontium carbonate (SrCO_3_), and copper carbonate (CuCO_3_) powders were mixed following the molar ratio of 4:1:1. The mixture was homogeneously grounded and put into a crucible to heat at 1000 °C for 16 h. The sintered mixture was re‐grounded and heated again following the same heating procedure. The as‐obtained raw SC powders were purified using a high concentration (1 m) hydrogen chloride (HCl) solution and washed with deionized (DI) water at least three times. To develop SC NSs, SC powders were exfoliated in DI water using high power (1000 W) ultrasonication for 6 h in an ice bath. SC NSs were centrifugated between 4000–8000 rmp for 15 min and collected for further use.

### Fabrication of 3D Printed SC/PCL Composite Scaffolds

SC/PCL composite scaffolds were fabricated using an extrusion‐based 3D printing technique. Briefly, PCL and SC (2, 4, and 8 wt%) were homogeneously mixed in chloroform and cast on the inner wall of the beaker to form a thin film. After the chloroform was evaporated, the SC/PCL composite film was then melted at 80 °C and transferred into the syringe for 3D printing (BioScaffolder 3.1, Gesim, Germany). The dosing pressure and applied temperature were 600–700 kPa and 90 °C, respectively, while the speed of the dispensing unit was 1–3 mm s^−1^ according to the concentration of the printing inks. Scaffolds of 50 mm × 50 mm × 1.5 mm following a crossed lay‐dawn pattern (45°) were prepared and cut into cylinders (diameter of 5 mm) for future use. As a control, pure PCL scaffolds were also 3D printed using the same protocol.

### Characterization of SC NSs and 3D Printed SC/PCL Composite Scaffolds

The morphology of SC powders was observed using a SEM (FEI APREO S, Thermo Scientific, Netherland). The X‐ray diffraction (XRD) patterns of SC powders were obtained using the Empyrean instrument (PANalytical, Netherlands). The morphology and thickness of SC NSs were characterized by a transmission electron microscope (TEM, JEM‐2100F, JEOL, Japan) and an atomic force microscope (AFM, Dimension Icon, Bruker, USA), respectively. The surface morphology and elemental analysis of scaffolds were conducted using SEM (FEI APREO S, Thermo Scientific, Netherland) and energy‐dispersive X‐ray spectroscopy (EDS). The photothermal performance of scaffolds was evaluated using NIR‐II laser (1064 nm) with different power densities (0.3–1.2 W cm^−2^). The released ions from SC/PCL composite scaffolds were assessed via an Avio 200 ICP‐OES System (PerkinElmer Inc., USA). The mechanical property of scaffolds was measured through an Instron machine (Instron‐5566, Instron, USA). For the accelerated degradation experiment, all the scaffolds were soaked in 5 m NaOH solution at 37 °C with bath shaking and weighed at day 1, 2, and 4.

### Effect of 3D Printed **SC/PCL** Composite Scaffolds on rBMSC Proliferation and Differentiation

rBMSCs were purchased from the Cell Bank (Chinese Academy of Sciences, Shanghai, China) and maintained in MEM*α* medium (GlutaMAX, Gibco, USA) supplemented with 1% penicillin/streptomycin and 10% fetal bovine serum (FBS). For cell proliferation, rBMSCs were seeded on 3D printed scaffolds in 48‐well plates with a cell density of 0.5 × 10^4^ cells/well, and cultured for 1, 3, and 7 days, respectively. The cell viability was evaluated using the CCK‐8 assay kit by determining the absorbance at 450 nm via a multifunction microplate reader (Bio‐Tec Instruments, USA). The cells cultured on scaffolds for 7 days were further stained using the ALP dye kit (Solarbio Life Sciences &Technology co., Ltd., Beijing, China) and the quantitative analysis of ALP expression was conducted using ALP assay kit and BCA assay kit (Pierce, Rockford, IL, USA), respectively. The effect of the scaffolds on osteogenic gene expression of rBMSCs was evaluated by qRT‐PCR. Briefly, 1 × 10^5^ cells were seeded on scaffolds in 6‐well plates for 7 days. The total RNA was extracted using Trizol reagent and transcribed into cDNA by a cDNA transcription kit (Invitrogen, USA). The obtained cDNA product was subsequently subjected to PCR amplification with multiplex primer sets related to the following osteogenic genes: osteocalcin (OCN), bone morphogenetic protein‐2 (BMP2), and RUNX2.

### Effect of 3D Printed SC/PCL Composite Scaffolds on HUVEC Proliferation, Migration, Tube Formation, and Differentiation

HUVECs were purchased from EK‐Bioscience (Shanghai, China) and maintained in endothelial cell medium (ECM, EK‐Bioscience, Shanghai, China) in an incubator with 5% CO_2_ atmosphere at 37 °C. For cell proliferation, HUVECs were seeded on 3D printed scaffolds in 48‐well plates with a cell density of 0.5 × 10^4^ cells/well and cultured for 1, 3, and 7 days, respectively. The cell viability was evaluated using the CCK‐8 assay kit. For cell migration testing, a typical transwell assay was applied. Briefly, 5 × 10^4^ cells were seeded on the upper chamber of transwell with serum‐free medium, while 3D printed scaffolds were placed in the bottom with the serum‐contained medium. After incubation for 12 h, the cells in the upper chamber were carefully removed and the migrated cells were fixed in 4% paraformaldehyde and stained with crystal violet (0.1% w/v) for visualization. For tube formation assay, a Matrigel‐coated plate was applied. 2 × 10^4^ cells/well were seeded in a 24‐well plate and co‐cultured with 3D printed scaffolds for 8 h. The formed tubes were observed by the microscope (DM2500, Leica Microsystems, Germany) and analyzed using ImageJ software (National Institutes of Health, USA). The effect of the scaffolds on angiogenic gene expression of HUVECs was evaluated by qRT‐PCR, following the same procedure as mentioned in Section 2.5 using the following genes: VEGF, hypoxia‐inducible factor‐1 alpha (HIF‐1*α*), and basic fibroblast growth factor (BFGF).

### Effect of 3D Printed SC/PCL Composite Scaffolds on Vascularized Bone Regeneration In Vivo

A rat critical‐sized cranial bone defect model was built according to a previous study.^[^ ^22^ ^]^ Briefly, Sprague Dawley (SD) rats were anesthetized by intraperitoneal injection of pentobarbital (Nembutal 3.5 mg/100 g), and two cranial defects with a diameter of 5 mm were created, followed by the subsequent implantation with 3D printed scaffolds. Male SD rats (6–7 weeks old) were purchased from Shanghai Jihui Laboratory Animals Care Co., Ltd. (Shanghai, China). All experiments were conducted following the guidelines of the Ethical Committee of Shanghai Ninth People's Hospital Affiliated with Shanghai Jiao Tong University School of Medicine. At the time point of 1 and 3 months, Micro‐CT imaging and histopathological analysis were conducted. Briefly, the defect sites were cut and fixed in 4% paraformaldehyde for 24 h. The new bone visualization was scanned and reconstructed by a Micro‐CT machine (Bruker micro‐CT, Kontich, Belgium). Quantitative analysis, including the new bone volume relative to tissue volume (BV/TV) and bone mineral density (BMD) was also performed. For histological and immunohistochemistry evaluations, the samples were decalcified in 10% EDTA for 4 weeks and then embedded in paraffin wax. Slices with a thickness of 4 µm were sectioned and stained with H&E and Masson's trichrome. Primary antibodies targeting osteopontin (OPN), RUNX2, platelet endothelial cell adhesion molecule‐1 (PECAM‐1, CD31), and HIF‐1*α* were employed for immunohistochemistry staining.

### In Vitro Anticancer Effects of Scaffolds

To investigate the in vitro anticancer effect of scaffolds, osteosarcoma cells (Saos‐2, EK‐Bioscience, Shanghai, China) were seeded on PCL and SC/PCL composite scaffolds in 48‐well plates (5 × 10^4^ cells/well) in an incubator (5% CO_2_, 37 °C) for 24 h. The culture medium (McCoy's 5A, HyClone, USA) supplemented with 1% penicillin/streptomycin and 15% fetal bovine serum (FBS) was used. Sequentially, NIR‐II laser (1064 nm) with different power densities (0, 0.3, 0.6, 0.9, 1.2 W cm^−2^) was applied for photothermal ablation. After 5 min laser irradiation, the cell viability was evaluated using the CCK‐8 assay kit. For live/dead cell staining, Saos‐2 cells seeded on different scaffolds were incubated in media containing calcein‐AM (5 µm) and PI (5 µm) for 20 min. Then, the cell/scaffold constructs were washed twice with PBS and observed using a confocal laser scanning microscope (Leica TCS SP5, Leica Microsystems, Germany).

### In Vivo Antitumor Effects of Scaffolds

The healthy Balb/c female mice (4‐6 weeks old) were purchased from Guangdong Laboratory Animal Co., Ltd. (Guangdong, China) and were used in accordance with the regulations of the Animal Ethical and Welfare Committee of Shenzhen University (AEWC‐SZU). The tumor‐bearing mice model was established by subcutaneously injecting 1 × 10^7^ Saos‐2 cells in the back of each mouse. When the tumor volume reached about 100 mm^3^, all the mice were randomly separated into 4 groups as implanted PCL scaffolds in tumor with/without NIR‐II laser irradiation (1 W cm^−2^, 5 min) and implanted SC/PCL scaffolds in tumor with/without NIR‐II laser irradiation (1 W cm^−2^, 5 min). The real‐time tumor temperature in vivo was monitored by a NIR thermal imaging system. Tumor volume (V) was measured every 2 days following the formula: *V* = length × width^2^/2. The tumor from each group was collected and stained with H&E and TUNEL. The major organs (heart, liver, spleen, lungs, and kidneys) harvested from each group after 14 days post‐treatment were also stained with H&E to determine antitumor therapeutic effects.

### Statistical Analysis

All results in this study were obtained from at least three duplicate samples and exhibited as mean ± standard deviation using one‐way ANOVA analysis. A value of *p* < 0.05 was considered statistically significant (* for 0.01 < *p* < 0.05, ** for 0.001 < *p* < 0.01, and *** for *p* < 0.001).

## Conflict of Interest

The authors declare no conflict of interest.

## Supporting information

Supporting Information

## Data Availability

Research data are not shared.
