# Supplementary material for: 3D Printed Wesselsite Nanosheets Functionalized Scaffold Facilitates NIR‐II Photothermal Therapy and Vascularized Bone Regeneration
Source: Adv Sci (Weinh). 2021 Aug 15;8(20):2100894. doi: 10.1002/advs.202100894 (PMC8529444; doi:10.1002/advs.202100894)
Supplement: Supplementary file 1 — Supporting Information [file ADVS-8-2100894-s001.pdf]

WILEY-VCH

© 2021 Wiley-VCH GmbH

ADVANCED SCIENCE
Open Access

Supporting Information

for *Adv. Sci.*, DOI: 10.1002/advs.202100894

3D printed wesselsite nanosheets functionalized scaffold facilitates NIR-II photothermal therapy and vascularized bone regeneration

Chen Yang, Hongshi Ma, Zhiyong Wang, Muhammad Rizwan Younis, Chunyang Liu, Chengtie Wu, Yongxiang Luo,* Peng Huang**

DOI: 10.1002/((please add manuscript number))
Article type: Full Paper

3D printed wesselsite nanosheets functionalized scaffold facilitates NIR-II photothermal therapy and vascularized bone regeneration

Chen Yang, Hongshi Ma, Zhiyong Wang, Muhammad Rizwan Younis, Chunyang Liu, Chengtie Wu, Yongxiang Luo,* Peng Huang**

Dr. C. Yang, Dr. M. R.Younis, Z. Wang, C. Liu, Prof. Y. Luo and Prof. P. Huang
Marshall Laboratory of Biomedical Engineering, International Cancer Center, Laboratory of Evolutionary Theranostics (LET), School of Biomedical Engineering, Shenzhen University Health Science Center, Shenzhen 518060, China.
E-mail: peng.huang@szu.edu.cn (P. Huang); luoyongxiang@szu.edu.cn (Y. Luo).

Dr. H. Ma and Prof. C. Wu
State Key Laboratory of High Performance Ceramics and Superfine Microstructure, Shanghai Institute of Ceramics, Chinese Academy of Sciences, 1295 Dingxi Road, Shanghai 200050, China.
E-mail: chengtiewu@mail.sic.ac.cn

Dr. C. Yang
Wenzhou Institute, University of Chinese Academy of Sciences, Wenzhou, Zhejiang 325000, China

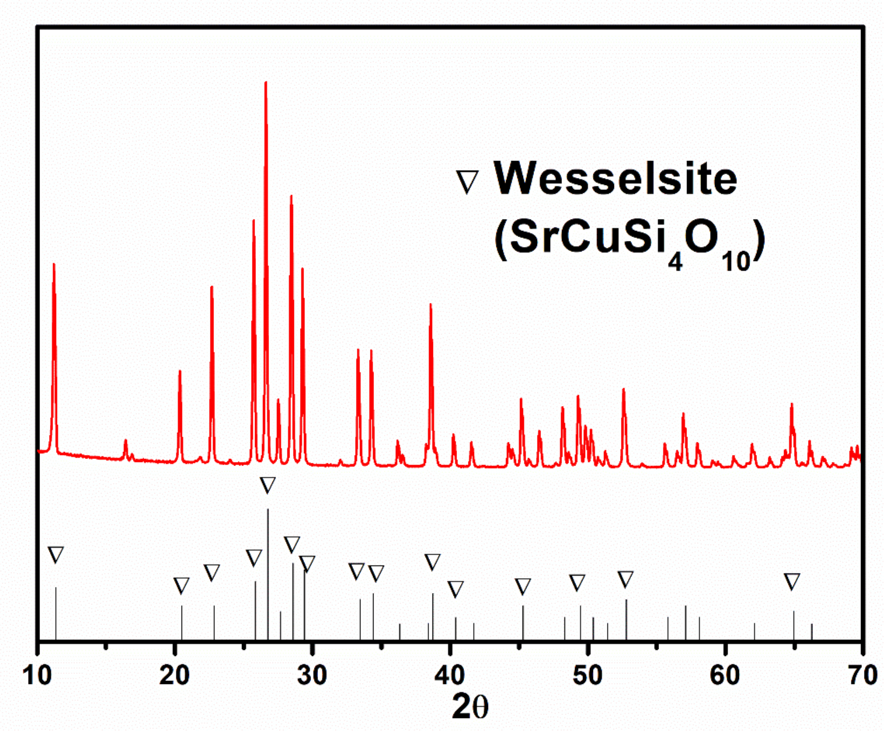

Figure S1. XRD patterns of SC powders.

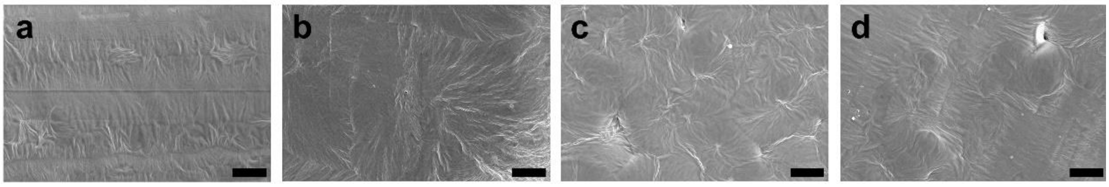

Figure S2. SEM characterization of the surface morphology of different 3D printed scaffolds. (a) PCL, (b) 2-SC/PCL, (c) 4-SC/PCL and (d) 8-SC/PCL. Scale bar: 5 μm.

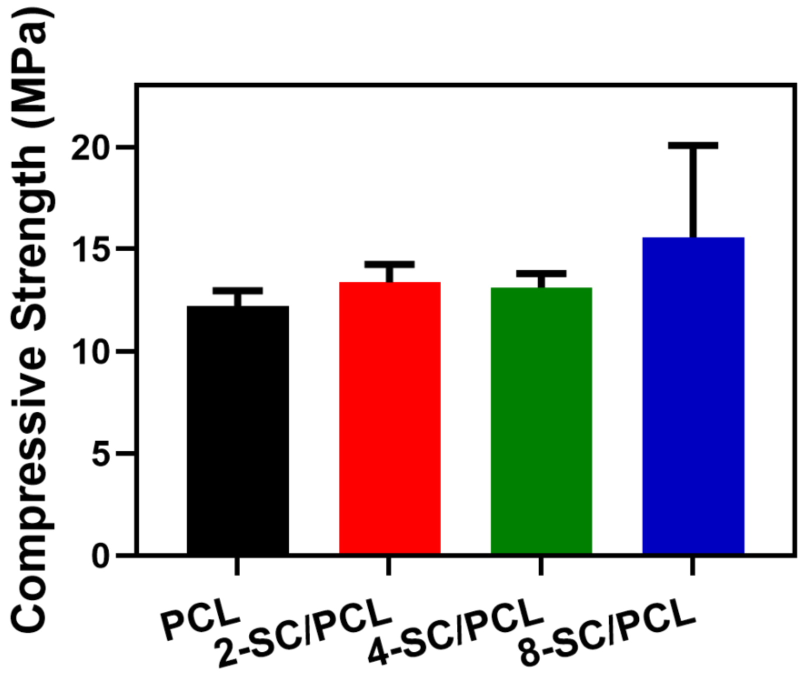

Figure S3. Compressive strength of different 3D printed scaffolds. Data are presented as mean ± s.d. (n = 3). One-way ANOVA analysis.

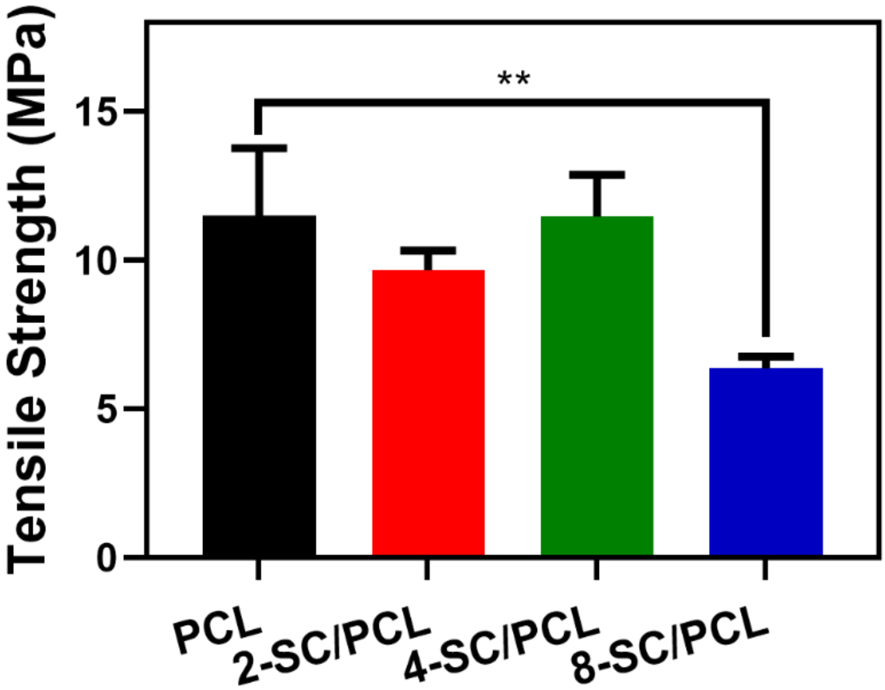

Figure S4. Tensile strength of different 3D printed scaffolds. Data are presented as mean ± s.d. (n = 3). ** for 0.001 < P < 0.01. One-way ANOVA analysis.

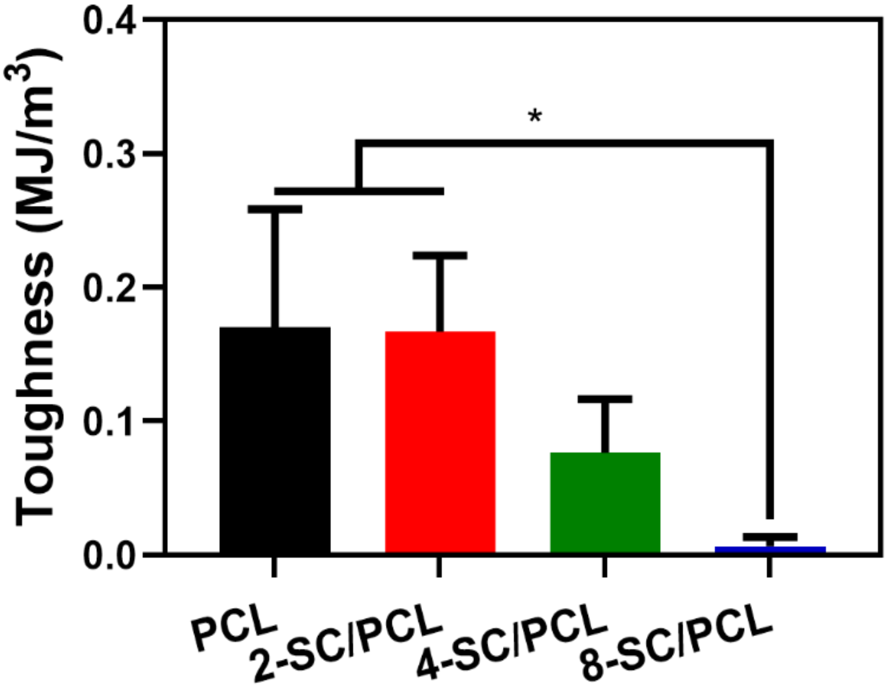

Figure S5. Toughness of different 3D printed scaffolds. Data are presented as mean ± s.d. (n = 3). * for 0.01 < P < 0.05. One-way ANOVA analysis.

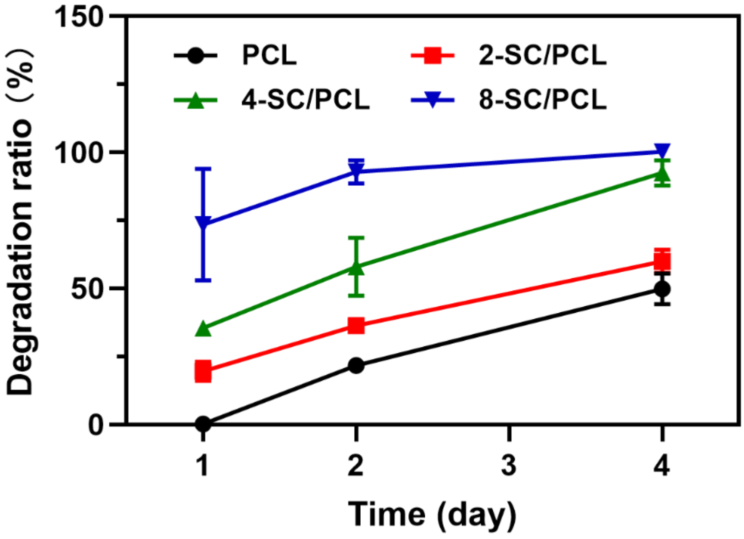

Figure S6. Quantitative analysis of scaffolds degraded in 5 M NaOH aqueous solution for 4 days. Data are presented as mean ± s.d. (n = 3).

	Before degradation	After degradation
Highest temperature (°C)	45.48 ± 0.97	41.77 ± 2.15
Compressive strength (MPa)	13.1 ± 0.69	7.53 ± 0.58
Compressive Young's modulus (MPa)	128.31 ± 3.22	73.45 ± 4.26

Table S1. The photothermal and compressive mechanical properties of 3D printed composite scaffolds (taking 4-SC/PCL as an representative scaffold) before and after degradation in PBS for 4 weeks. Data are presented as mean ± s.d. (n = 3).

	2-SC/PCL	4-SC/PCL	8-SC/PCL
**Sr** (ppm)	0.267 ± 0.029	0.776 ± 0.05	1.992 ± 0.283
**Cu** (ppm)	0.459 ± 0.063	1.492 ± 0.239	4.044 ± 0.416
**Si** (ppm)	5.384 ± 0.855	13.339 ± 1.839	24.78 ± 3.922

Table S2. The ionic concentrations released from different 3D printed composite scaffolds in cell culture media for 3 days. Data are presented as mean ± s.d. (n = 4).
